# Identifying symptom communities and core symptoms in the anxiety-depression network among computer science students

**DOI:** 10.1038/s41598-026-39553-w

**Published:** 2026-04-08

**Authors:** Wei Yi, Kun Yang, Zhengfan Wei, Mohd Mahzan Awang, Wan Ahmad Munsif Wan Pa, Yonglin Chen, Meiyang Wang, Shuoyu Jing

**Affiliations:** 1 College of Information Engineering, Zhengzhou University of Science and Technology, Zhengzhou, China; 2 Faculty of Education, National University of Malaysia, Bangi, Malaysia; 3 College of Clinic, Sanquan College of Xinxiang Medical University, Xinxiang, China

**Keywords:** Anxiety, Depression, Psychological network analysis, Computer science students, Symptoms community, Psychology, Health care

## Abstract

Although the mental health of college students has become a focus in the health field and society, there is still little discussion about the mental health of students in computer science and related majors. This study was guided by the network theory of mental disorders, presents a symptom network analysis of anxiety and depression among computer science students. A total of 3934 computer science students were included in this study. The seven-item Generalized Anxiety Disorder Scale (GAD-7) and the nine-item Patient Health Questionnaire (PHQ-9) were used to measure anxiety and depression symptoms. The connection between Nervousness and Uncontrollable worry is the strongest edge in the network. We identified the three core symptoms with the highest node strength were concentration, fatigue and psychomotor problems. The three bridge symptoms with the highest bridge strength were irritability, feeling afraid and psychomotor problems. Four well-characterized symptom communities were identified through the SpinGlass algorithm, including the core anxiety symptom community, the anxiety somatization manifestation symptom community, the core depressive symptom community, and the depressive physiological manifestation symptom community. The network performed well in both stability and accuracy tests. These findings are important for future interventions and improving the role of mental health issues for students with diverse majors and stressors.

**Supplementary Information:**

The online version contains supplementary material available at 10.1038/s41598-026-39553-w.

## Introduction

The mental health issues among university students have become a globally significant social concern, particularly in the context of today’s high-pressure societal and educational environments^1,2^. During their higher education journey, students face immense pressures from multiple dimensions, including academic demands, social adaptation and career development^3–5^. These complex stressors often lead to a dramatic increase in mental health issues such as anxiety and depression. Previous research indicates that the overall situation of depression among Chinese university students remains a public health concern, with a prevalence rate of 28.4%^6^.

Mental sub-health issues among STEM students have received increased attention over the past decade^7^. A famous study showed that nearly half of STEM graduate students experienced depression symptoms from the University of California, Berkeley^8^. Computer science and related majors are especially at high risk for severe mental health issues, with students in these fields reporting the highest levels of panic and anxiety^9,10^. Evidence from Brazil suggests that computer science students face even more anxiety and depression than medical students^11^, which traditionally seen as a high-risk group. Furthermore, empirical data found that computer science majors have twice the rate of anxiety and depression as the general undergraduate population^10^. This underscores the serious mental health challenges faced by students in computer-related fields. However, research on the mental health of STEM students, especially computer science and related majors, is still very limited in China.

In the context of increased competition in Chinese education, mental health problems among STEM students led by computer science majors, are likely to become a focus in the future. According to the authority’s 2024 undergraduate discipline rankings, the top 10 includes six stem-related disciplines such as computer science and software engineering^12^. However, behind this popularity majors and intensified competition lies the enormous pressure faced by students. Educational involution posing potential threats to their mental health^13,14^. As a representative discipline, computer science exemplifies the broader challenges faced by STEM programs under the backdrop of educational competition and involution in China. This pressure extends beyond academic demands to encompass employment preparation and career development^15,16^, further exacerbating the anxiety on students.

Multiple studies have shown that, in addition to academic and social factors, computer anxiety has emerged as a critical factor affecting the mental well-being of students^17,18^. Research indicates that this anxiety or technophobia often stems from a lack of confidence or excessive worry about their skills of computer technologies^19,20^. Research has shown that anxiety significantly affects students’ attitudes toward learning and academic performance, thereby negatively affecting their mental health^19,21^. The rapid development and updating iterations of information technology have made computer anxiety and technophobia a common problem for computer science major students^11^. Additionally, individuals are more likely to suffer from mental health problems when they are in a digital work and learning environment for a prolonged period of time^22^. Such environments can lead to a wide range of psychological distress, including career anxiety, technological stress, and burnout^23^.

To deeply investigate the characteristics of anxiety and depression problems in the Chinese computer science student population, this study used psychological network analysis. The network theory of mental disorders proposed by Borsboom suggests mental disorders to be systems of interactions between symptoms^24^. This theoretical framework suggests that various psychological symptoms are not only manifested in results but may also actively interact with each other to maintain the entire symptom network structure^24,25^. Therefore, the use of network analysis to identify the core and bridging symptoms that sustain anxiety-depression network has important theoretical support and practical significance. By constructing a psychological symptom network model, the core symptom nodes of anxiety and depression and their interactions can be systematically examined from a network perspective^26^. As an innovative tool for studying the relationships between nodes in complex networks or systems, network analysis has been widely used in recent years in psychopathology and psychology^24,27^. It has also been applied across various students’ populations, including medical students, nursing students and student-athletes, to explore the structure and interaction of psychological symptoms within specific contextual stressors^28–30^. Individual psychological symptoms are conceptualized as nodes in a network, while interactions between symptoms are characterized by edges between nodes^24^. Focusing on visualizing the overall structural features of the psychological symptom system, as well as the centrality of specific symptoms in the network and their potential impact on the overall system^31^. This approach provides a more comprehensive anxiety and depression network research perspective. At the micro level, it evaluates the role of individual symptoms within the network, such as strength, bridge strength, predictability and expected influence^32,33^. At the meso level, community detection methods identify interaction patterns among symptom clusters^34^. At the macro level, global features such as network stability and differences across networks can be estimated^35^. Network analysis plays a critical role in symptom identification within mental health studies and offers valuable insights for clinical intervention and prevention^36^.

Although an increasing number of studies have explored anxiety and depression among Chinese university students from a network perspective, most of these studies focus on medical-related groups^37,38^. To date, no network analysis has been identified that specifically examines anxiety and depression among undergraduate computer science students. Previous evidence has already highlighted the concerning mental health conditions of computer science students, yet this group remains underexplored, particularly within the context of China’s educational environment. This study aims to conduct a network analysis of anxiety and depression among Chinese undergraduate computer science students. The primary goal of this research is to investigate the structure, community and stability within the anxiety and depression networks of this population, providing a foundation for developing tailored psychological interventions for this specific group in the future.

## Methods

### Participants & procedure

This study is a large investigation into the anxiety and depression networks of undergraduate computer science students in China. Given the research team’s available resources, participant recruitment was conducted among undergraduate computer science students in Henan Province, China. A total of 3934 computer science students were included in this study. Data were collected via an online survey distributed through the Wenjuanxing platform (https://www.wjx.cn). Eligibility criteria for participants included the following: (a) above18 years old, (b) undergraduate students and (c) their major must belong to the category of computer-related disciplines as defined in the Undergraduate Major Directory of General Higher Education Institutions published by the Ministry of Education of China (http://www.moe.gov.cn/). The ‘computer science’ major in this study include computer science and technology, software engineering, network engineering, information security, artificial intelligence, and other majors belonging to the category of computing disciplines in China. The study received approval from the Academic Committee of Zhengzhou University of Science and Technology. All methods were performed in accordance with the relevant guidelines and regulations. All participants provided online written informed consent before completing the questionnaire. Data collection took place from December 16, 2024, to January 15, 2025.

## Measures

The anxiety symptoms was measured using the Chinese version of the 7-item Generalized Anxiety Disorder Scale (GAD-7)^39^, with item scores ranging from 0 (not at all) to 3 (nearly every day) and a total score range of 0 to 21. Anxiety severity is classified as minimal (1–4), mild (5–9), moderate (10–14), and severe (15–21). The depression symptoms was assessed using the Chinese version of the 9-item Patient Health Questionnaire (PHQ-9)^40^, with item scores ranging from 0 (not at all) to 3 (nearly every day) and a total score range of 0 to 27. The depression severity is classified as minimal (1–4), mild (5–9), moderate (10–14), moderately severe (15–19) and severe (20–27).The reliability and validity of the Chinese version on anxiety and depression scales have been confirmed in previous studies^41,42^.

### Data analysis

All analyses were performed by using the R^35^. The network analysis was performed in three parts including network estimation, network stability and network community detection.

Network estimation. This study constructed a psychological symptom network model by treating symptoms as nodes and their interrelationships as edges. To accurately estimate the strength of associations between nodes, partial correlation analysis was employed to calculate conditional dependencies between symptoms while controlling for the influence of other nodes. To deal with the variability and potential non-normality of scores from the GAD-7 and PHQ-9 scales, we applied a nonparanormal transformation function from the huge R package. This rank-based and non-parametric approach maps the empirical cumulative distribution of each variable to a standard normal distribution, thereby standardizing the data while preserving the ordinal relationships^43^. The partial correlation matrix is provided in supplementary material. To enhance the interpretability of the network, the Least Absolute Shrinkage and Selection Operator (LASSO) regularization^44^ was implemented to simplify the network structure by shrinking small edge weights toward zero, thereby obtaining a sparse and more interpretable network model. Using the Extended Bayesian Information Criterion (EBIC) optimization and tuning parameters is to ensure that the network is sparse and interpretable^45^. Visualization and estimation is carried out by using the bootnet and qgraph packages in R^35^. Edge thickness indicates the strength of the connection between nodes. Red represents negative correlations and green represents positive correlations. The core metrics are obtained through the networktool and qgraph package, include strength, bridge strength and expected influence^46^. Some of the metrics are not suitable for usage in all research contexts and require additional attention, such as betweness and clonesness^47^. Predictability quantifies how much of a node’s variance can be explained by its neighboring nodes^45^. Bridging nodes mainly identify the nodes with connectivity role in the symptoms network, bridge strength is an important indicator to measure bridge nodes. Bridge strength refers to the sum of edge weights between a given node and nodes in other communities, which was analyzed using the bridge function of the R package networktools^36^. Expected Influence is used to measure the combined influence of a node on other nodes in the network, considering both strength and direction. Strength, defined as the sum of the absolute edge weights connected to a node, reflects the overall level of symptom connectivity^24^. While expected influence is more appropriate when there are certain negative edges in the network, strength provides a more stable and interpretable centrality measure for identifying core symptoms in networks primarily composed of positive edges.

Network stability. All network stability analyses are performed with the bootnet package (Version 1.4.3)^48^. In this study, the stability of node strength and bridge strength was assessed using a case-dropping bootstrap procedure. This is to ensure that the network metrics remain reliable and generalizable after some of the data are deleted^35^.. The study utilized the Correlation Stability Coefficient (CS-C) to quantify network stability, which indicates the maximum proportion of cases that can be removed while maintaining a correlation of at least 0.7 with the original network’s centrality indices. According to academic standards, a CS-C value above 0.25 is considered acceptable, while values exceeding 0.5 indicate high network stability^35^. Furthermore, a nonparametric bootstrap procedure was implemented to calculate 95% confidence intervals (CIs) for edge weights and node strength to assess estimation precision, where narrower confidence intervals signify higher estimation accuracy^35,49^.

Network community. The SpinGlass algorithm was used for community segmentation of anxiety-depression symptom networks. The algorithm has been shown to be effective for community detection by optimizing modular functions to identify densely connected symptom clusters^50,51^. Community detection not only reveals patterns of interaction between symptoms, but also identifies differences in symptom clusters across groups, providing important insights into understanding patterns of co-occurrence of psychological symptoms^52^. This algorithm has been successfully applied to analyze the network structure of post-traumatic stress disorder (PTSD) symptoms in Chinese male firefighters, and its validity and applicability have been verified^53^.

**Table 1:** Demographic of participants.

Year of undergraduate study		*N* (%)
	First	1191 (30.27)
	Second	843 (21.43)
	Third	1172 (29.79)
	fourth	728 (18.51)
Gender		
	Male	2210 (56.17)
	Female	1724 (43.83)
First-generation college student in family		
	Yes	2272 (57.75)
	No	1662 (42.25)
Residential area		
	City	709 (18.02)
	Rural	3225 (81.98)

## Results

### Descriptive statistics

**Fig. 1:**
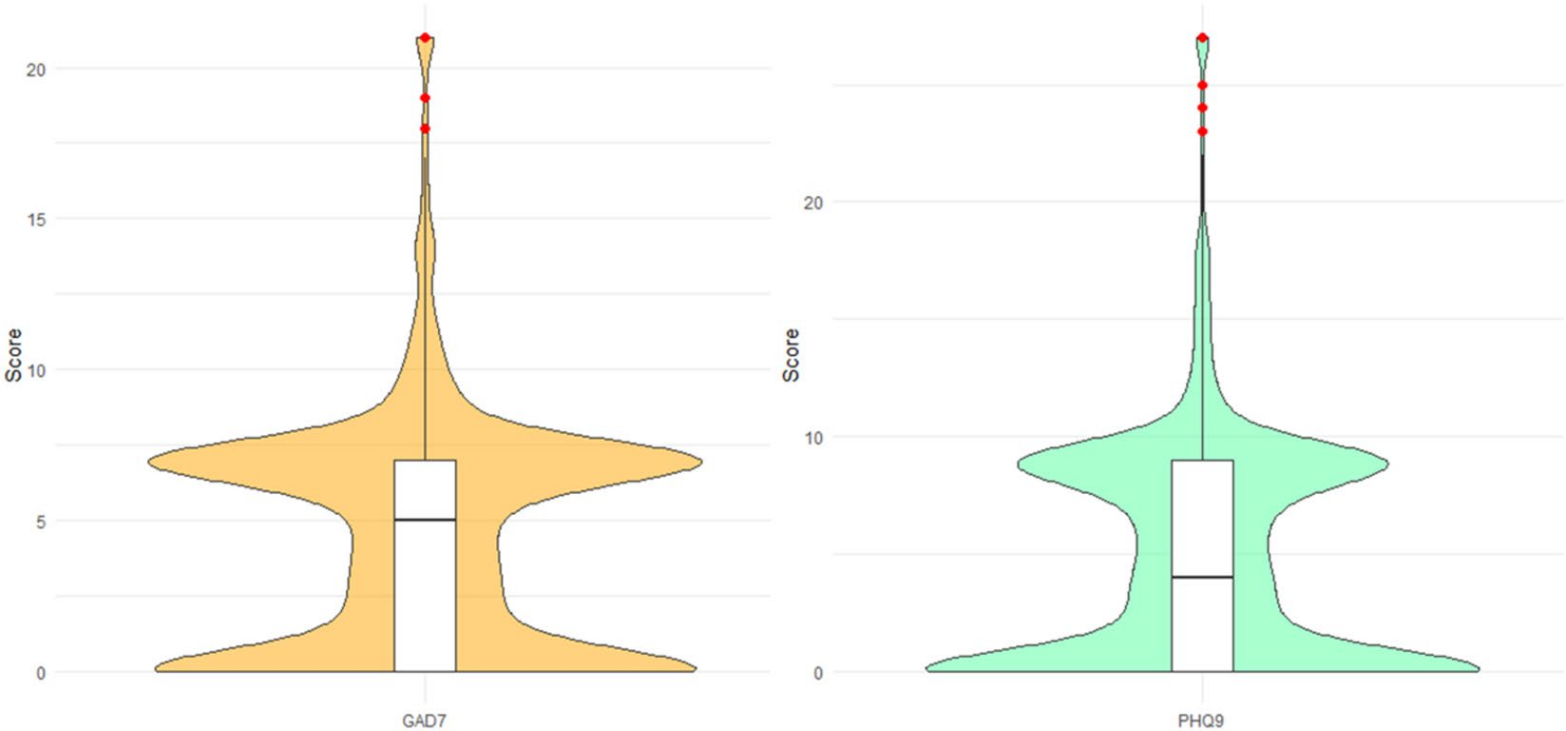
Distribution of GAD-7 and PHQ-9 scores.

Participant demographic information is shown in Table 1. The distribution of participants’ anxiety and depression scores based on GAD-7 and PHQ-9 is shown in Fig. 1.

In the GAD-7, 49.12% of the participants were at no or minimal anxiety level and 44.77% were at mild anxiety level. 4.22% of the participants were at moderate level of anxiety and 1.88% were at severe level of anxiety. In the PHQ-9, 52.58% of the participants were at no or minimal level of depression and 37.99% were at mild level of depression. 6.20% and 2.08% of the participants were at moderate and moderately severe levels of depression, 1.14% were at severe levels.

### Network structure

**Fig. 2:**
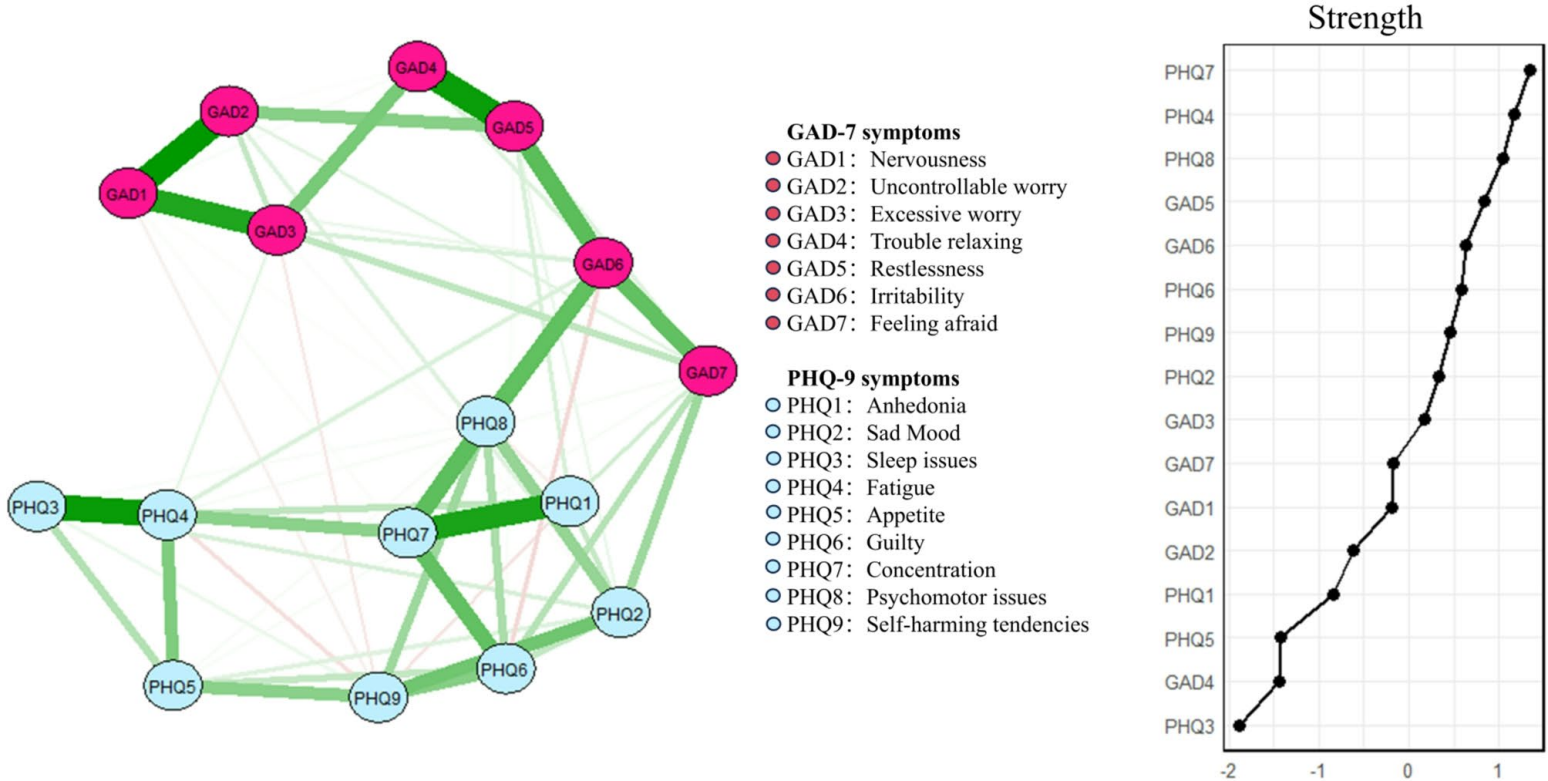
Network structure of anxiety and depressive symptoms among computer science students.

In the anxiety and depression network as shown in Fig. 2, the strongest edge was the Nervousness-Uncontrollable worry (GAD1-GAD2). In addition, Sleep issues-Fatigue (PHQ3-PHQ4), Trouble relaxing-Restlessness (GAD4-GAD5), Anhedonia-Concentration (PHQ1-PHQ7), Nervousness-Excessive worry (GAD1-GAD3) also showed strong associations. These connections represent the most prominent symptom pairings within the network.

**Fig. 3:**
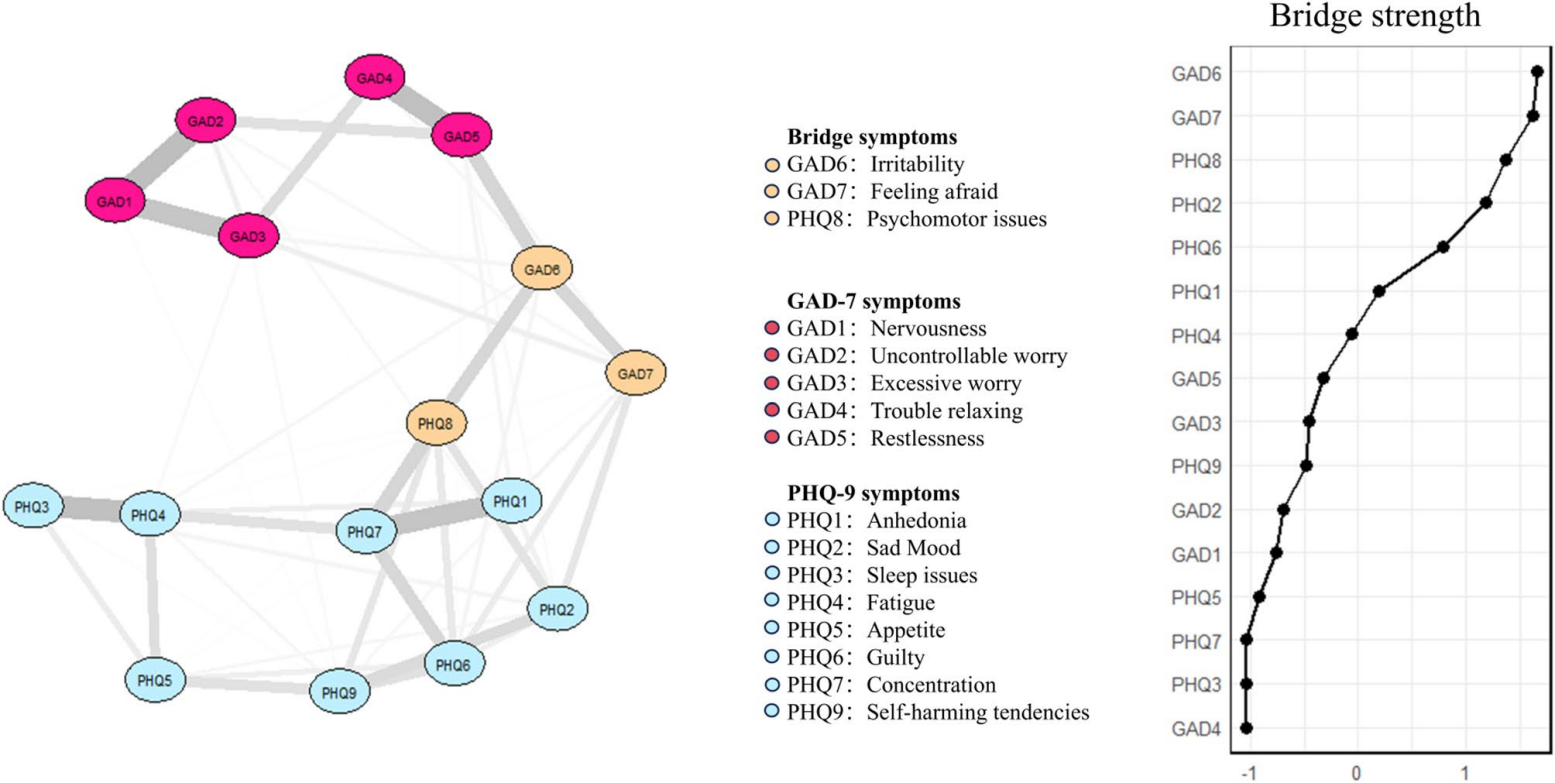
Network structure of anxiety and depressive symptoms present bridge symptoms among computer science students.

In Fig. 2; Table 2, Concentration (PHQ7) presented the strongest node strength, followed by Fatigue (PHQ4) and Psychomotor issues (PHQ8). Concentration (PHQ7) presented the strongest expected influence, followed by Restlessness (GAD5) and Psychomotor issues (PHQ8). Restlessness (GAD5) showed the strongest predictability, followed by Feeling afraid (GAD7) and Psychomotor issues (PHQ8). In Fig. 3, Irritability (GAD6) presented the strongest bridge strength, followed by Feeling afraid (GAD7) and Psychomotor issues (PHQ8).

## Network stability

**Fig. 4:**
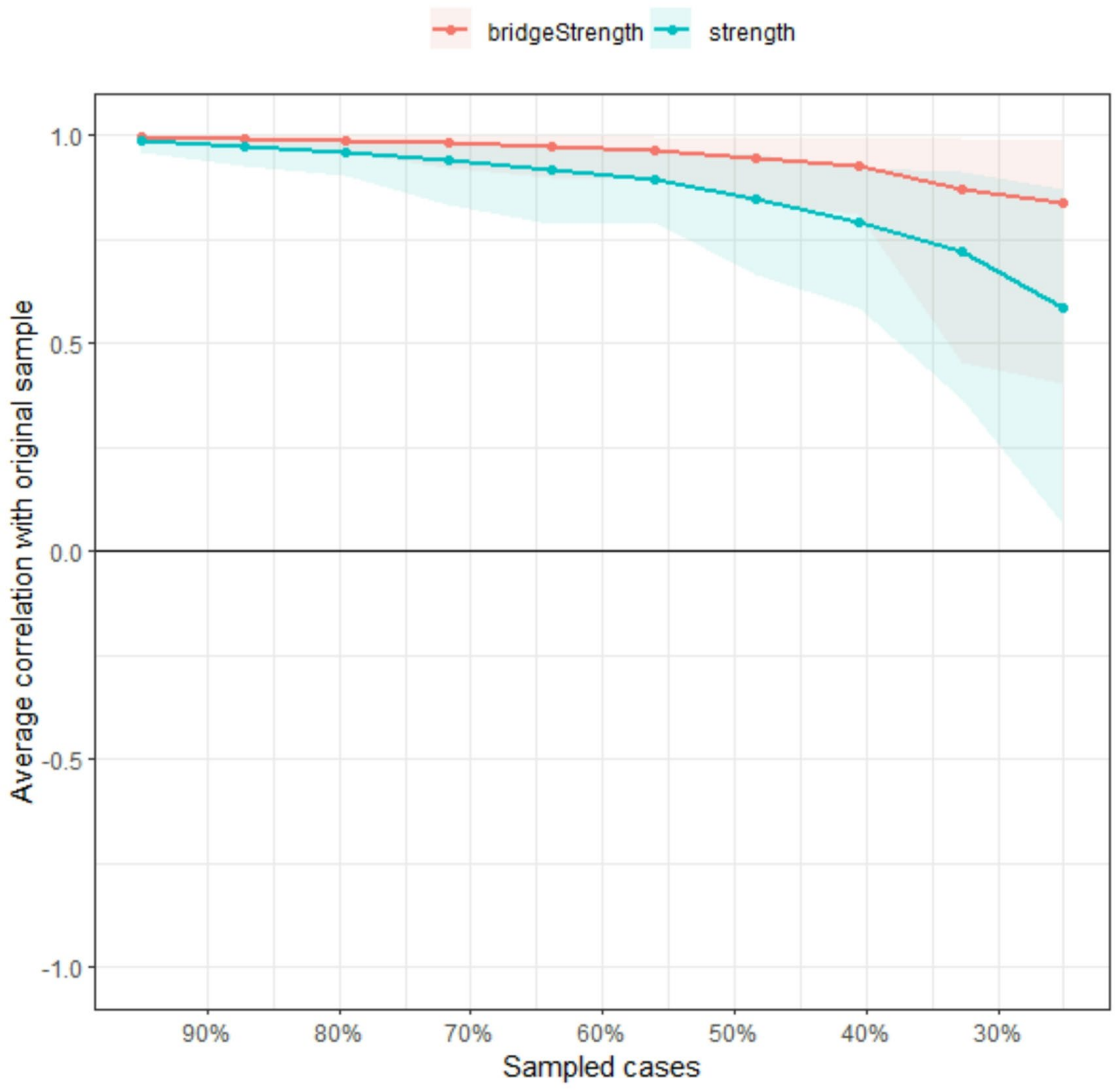
The network stability test of strength and bridge strength by using case-dropping bootstrap. The horizontal axis represents the proportion of the original sample retained, and the vertical axis represents the average correlation coefficient between the initial network index and the recalculated centrality index after case culling.

**Fig. 5:**
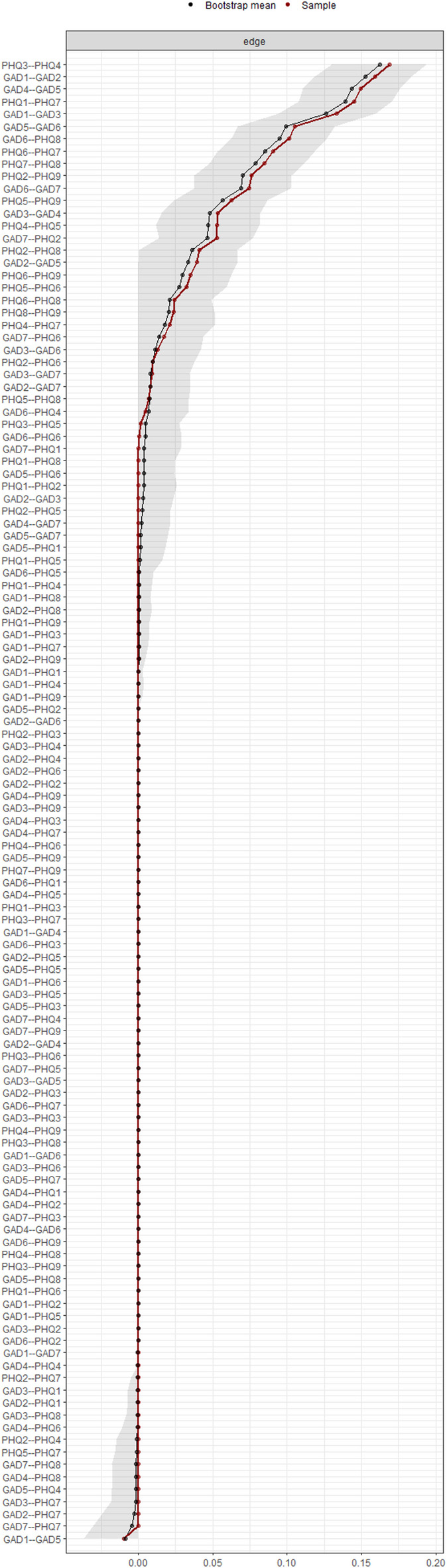
Bootstrapped confidence intervals of all edges. The black dots show ordered edge weights, from highest to lowest value. The surrounding gray area indicates the 95% confidence interval derived using the nonparametric bootstrap method. The wider the interval, the lower the stability. The narrower the interval, the higher the reliability of the estimated edge weights.

**Fig. 6:**
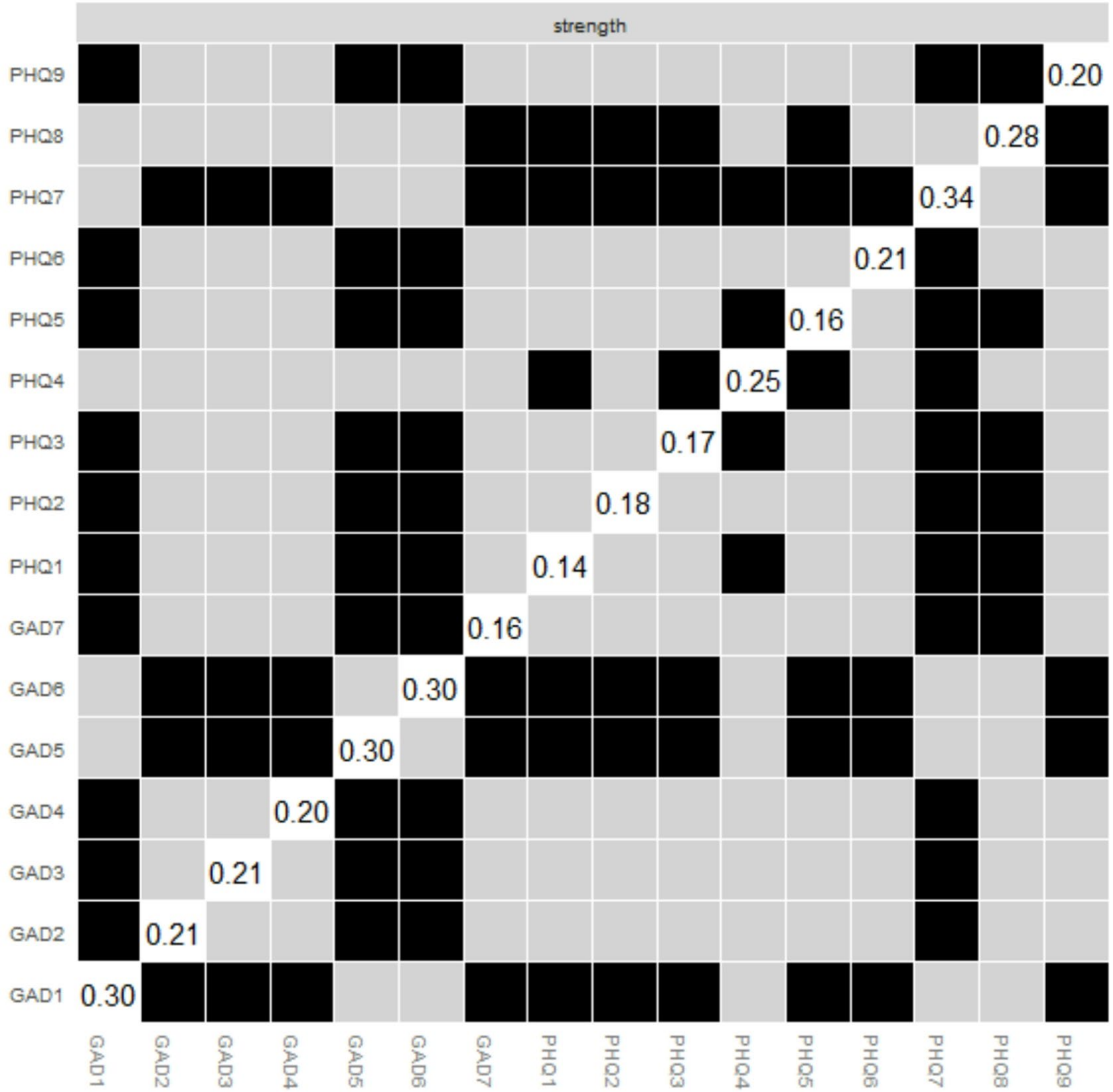
The stability test for ‘node strength’ based on Bootstrap. Gray boxes indicate no significant differences and black boxes indicate significant differences. Numbers in white boxes (i.e., diagonal lines) indicate node strength values for specific nodes.

In Fig. 4. The stability of the anxiety-depression network was evaluated by using case-dropping bootstrapping with the bootnet package in R. Both CS-C values exceed the recommended threshold of 0.5, indicating that the network metrics are stable under case-dropping conditions. The visual representation shows a gradual decline in the average correlation with the original sample as more cases are removed, with bridge strength demonstrating slightly higher stability compared to strength. Additionally, to assess the difference between two edges or two node strengths, a nonparametric bootstrap test based on 95% CI was performed. Figures 5 and 6 showed the results of bootstrapped confidence intervals in edges and bootstrap difference in nodes strength.

**Table 2:** Descriptive statistics of the PHQ-9 and GAD-7 items.

Item	Content	Item mean (SD)	Strength	Predictability	Expected Influence
GAD1	Nervousness	0.74 (0.65)	0.426	0.649	0.401
GAD2	Uncontrollable worry	0.59 (0.65)	0.395	0.693	0.395
GAD3	Excessive worry	0.69 (0.67)	0.451	0.673	0.410
GAD4	Trouble relaxing	0.61 (0.66)	0.337	0.691	0.337
GAD5	Restlessness	0.58 (0.63)	0.497	0.759	0.497
GAD6	Irritability	0.60 (0.64)	0.483	0.715	0.423
GAD7	Feeling afraid	0.57 (0.63)	0.426	0.734	0.426
PHQ1	Anhedonia	0.65 (0.69)	0.379	0.646	0.308
PHQ2	Sad Mood	0.49 (0.61)	0.461	0.721	0.455
PHQ3	Sleep issues	0.58 (0.68)	0.306	0.603	0.306
PHQ4	Fatigue	0.63 (0.67)	0.521	0.717	0.472
PHQ5	Appetite	0.48 (0.62)	0.339	0.628	0.339
PHQ6	Guilty	0.51 (0.64)	0.479	0.715	0.420
PHQ7	Concentration	0.59 (0.65)	0.532	0.720	0.532
PHQ8	Psychomotor issues	0.51 (0.62)	0.512	0.733	0.483
PHQ9	Self-harming tendencies	0.31 (0.55)	0.470	0.505	0.319

GAD-7 7-item Generalized Anxiety Disorder Scale, PHQ-9 the 9-item Patient Health Questionnaire, SD standard deviation

Edge colors are omitted to avoid color mixing.

## Network community

**Fig. 7:**
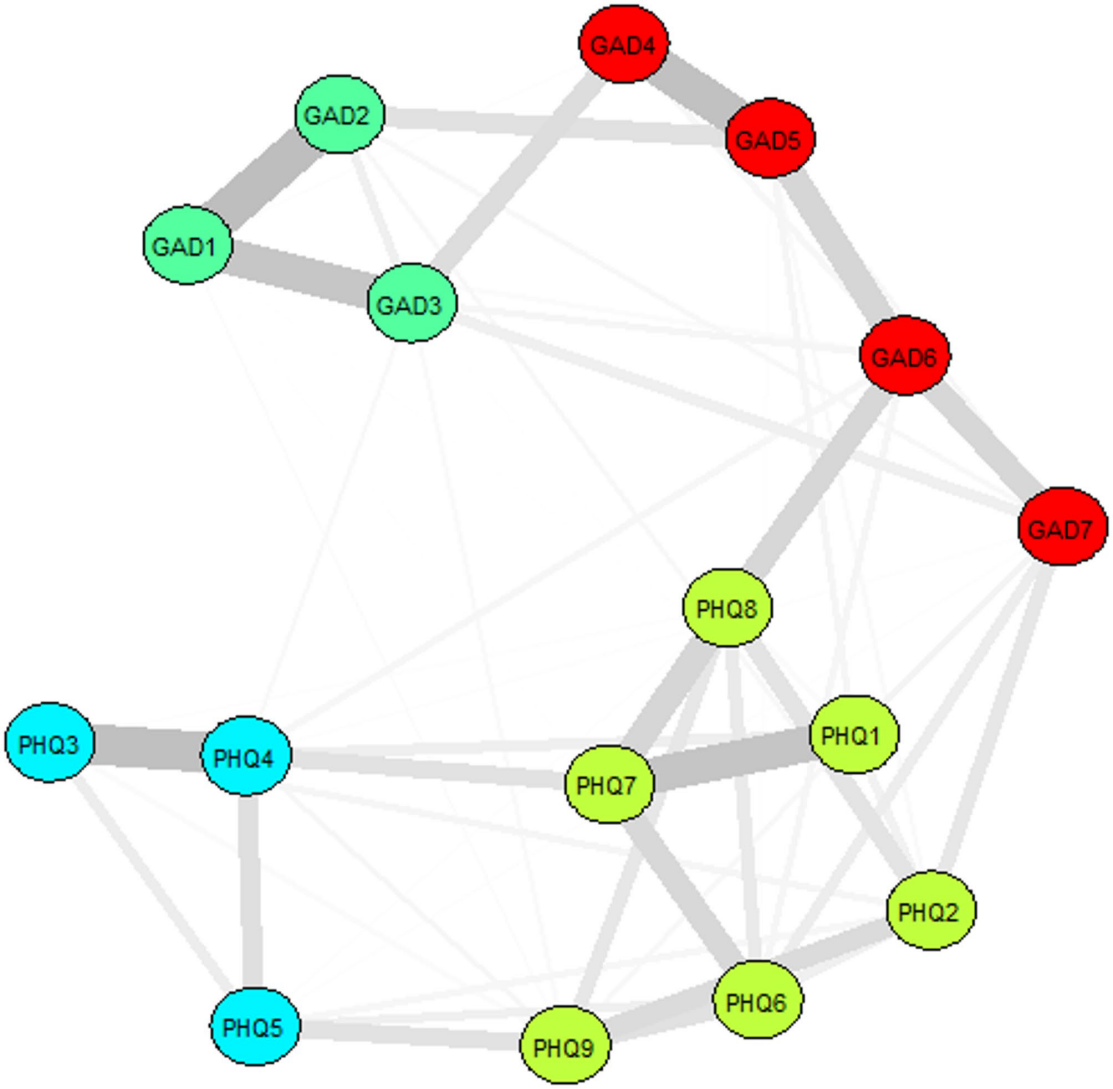
Community Detection Visualization. Different colors represent different communities. Edge colors are omitted to avoid color mixing.

Using the Spin Glass algorithm, four distinct communities were identified within the anxiety-depression network as shown in Fig. 7. The first community consists of Nervousness (GAD1), Uncontrollable worry (GAD2) and Excessive worry (GAD3). The second community includes Trouble relaxing (GAD4), Restlessness (GAD5), Irritability (GAD6) and Feeling afraid (GAD7). The third community comprises Anhedonia (PHQ1), Sad Mood (PHQ2), Guilty feelings (PHQ6), Concentration issues (PHQ7), Psychomotor issues (PHQ8) and Self-harming tendencies (PHQ9). The fourth community comprises Sleep issues (PHQ3), Fatigue (PHQ4) and Appetite (PHQ5).

## Discussion

As of the commencement of this study, this is the first article to conduct a network analysis of anxiety and depression among Chinese computer science students. Although the self-reported prevalence of clinically depression level around 10% among Chinese computer science students was lower than that observed in Chinese medical students’ level around 20%^54^, approximately 50% of participants in our study reported symptoms at or above the mild level. This means the mental health status of Chinese computer science students in terms of anxiety and depression deserves greater attention, and generalized anxiety and depression problems were also found among Brazilian computer science students^11^. Although computer science students are not traditionally considered a high-risk group, the findings of this study suggest that they experience similar challenges related to anxiety and depression as those observed in medical or healthcare student populations, such as student nurses and medical students^37,54^. This means the focus of mental health research and intervention should be beyond traditionally recognized high-risk groups. All the strongest edges in this study were within their respective symptom ranges and did not connect anxiety and depression symptoms, which is like previous findings^37,54,55^.

In previous studies on anxiety and depression networks among Chinese university students, the stronger edges were present within the anxiety cluster^55^. However, the second strong edge in the current study showed an association within the depression cluster, which is similar to the results of the previous study on nursing students^30^. This difference further suggests that variations in academic background may contribute to differing levels of depression and anxiety among students^56^. Factors such as computer anxiety and technophobia have been shown to affect students’ physical and mental health^17,18,23,57^. Technology anxiety presents itself particularly in technology-intensive professions, so future psychological interventions should also focus on supporting both emotional and technological self-confidence. Moreover, China’s extremely introspective educational environment is one of the external stressors that contribute to increased anxiety and depression among college students^13^. In addition to the strongest association Nervousness-Uncontrollable worry (GAD1-GAD2), Sleep issues-Fatigue (PHQ3-PHQ4) showed the second strongest symptomatic association, and this association was also high in several previous studies^30,55^. Sleep problems have been a common problem among college students^58^, and not just among computer science students. Improving sleep quality and increasing physical activity are considered effective intervention strategies^11^, have been suggested to alleviate depression and anxiety problems among computer science students.

Node strengths in network metrics are recognized by psychopathology and psychology as important potential targets for network intervention^37,49^. This study found that Concentration (PHQ7) was the symptom with the highest node strength in the Anxiety and Depression network for computer science students, followed by Fatigue (PHQ4). This is different from previous findings in other studies, where fatigue was typically the highest strength core symptom among college students^55^. This discrepancy may stem from the unique stressors and learning patterns experienced by computer science students. With the rapid development of artificial intelligence technology, students in computer-related fields face unprecedented pressure from industry updates and academic challenges^59^. The constant updating of technology and knowledge imposes an ongoing learning burden, where cognitive stress or overload is closely linked to anxiety^60^. Additionally, the prolonged screen-time work pattern not only leads to visual fatigue but may also trigger a series of physical and mental health issues^23^. Recent studies indicate that sedentary behavior and screen time among college students show significant negative correlations with mental health status, particularly in relation to depression and anxiety^57^. The high score for concentration (PHQ7) in terms of expected influence further suggests that it is a potential monitoring node in a dynamic network containing negative correlations. In contrast to focusing on the direct strength of the symptom, the expected influence provides a perspective to identify important symptoms for further clinical diagnostic assistant and targeted assessment, especially when no causal inference is formed^25^. Compared to traditional centrality that do not distinguish between positive and negative relationships, monitoring or targeting high expected influence symptoms is a viable strategy to help understand dynamic trends in the symptom network.

Irritability (GAD6) was identified as the most influential bridge symptom, followed by Feeling afraid (GAD7) and Psychomotor issues (PHQ8). The composition of the most influential bridge symptoms was like the previous results for the college student population^55,61^, but with a slightly different ordering. Intervening in bridging symptoms may offer a strategic advantage over direct intervention in high- strength symptom nodes, as it can prevent the spread across symptom clusters and potentially mitigate the escalation of comorbid conditions^62^. Psychomotor issues (PHQ8) have consistently demonstrated high bridge strength in pandemic-related studies^30,55,61^. This phenomenon may be attributed to the symptom’s strong association with self-harm tendencies and death ideation, making it a priority focus in clinical interventions^37^. These findings suggest that future intervention strategies should be tailored according to the transmission mechanisms and clinical priorities of different symptoms. It is important to note that although self-harming tendencies (PHQ9) had the lowest mean score among all dimensions, it warrants heightened attention due to its potential for severe consequences. Given the high bridge strength of PHQ-8 and its established association with PHQ-9, close monitoring and early intervention are essential when such symptoms arise.

By calculating the predictability of each symptom node in the network, this study revealed complex inter-symptom prediction patterns. High predictability values for symptoms such as Restlessness (GAD5), Feeling afraid (GAD7), and Psychomotor issues (PHQ8) suggest that these symptoms can be highly predicted based on their neighboring nodes. On average, 68.1% of the variance in each symptom could be predicted from its immediate neighbors. However, it should be noted that predictability does not imply causation, and it indicates how well a symptom can be predicted and explained from its neighbors’ nodes^45,63^.

Through community detection with the SpinGlass algorithm on the anxiety-depression network, it was found that the network can be divided into four separate communities, each containing at least one set of symptom clusters with highly correlated edges. This community structure suggests that associations between different symptoms are not randomly distributed but are concentrated in specific sub-clusters. Specifically, the green community reflects the core symptoms of anxiety, while the red community reflects the somatization and emotional manifestations of anxiety. The yellow community represents the core symptoms of depression, while the blue community is associated with the somatization of depressive symptoms. The results of this community detection provide a structured framework for future interventions, enabling the identification of key clusters and prioritizing the most densely connected and critical communities. Combining symptom communities with core symptoms allows for more accurate targeting, and early identification of potential symptoms for intervention in a more organized manner^64^. This approach no longer relies solely on apparently salient or obvious symptoms, such as insomnia in the current results, which may not play a central role in maintaining the symptom network. Different communities may require tailored intervention or treatment strategies based on their unique symptom profiles^53^. The personalized networks have become a reliable ‘psycho-educational’ tool to help patients and clinicians increase their awareness of symptom network problems and triggers^65^. Furthermore, in addition to identifying symptom clusters, community detection results can be used to predict potential transmission pathways for anxiety and depressive symptoms^66^. For example, in the case of suicidal tendencies with serious consequences, we can create risk assessment and prevention measures for specific symptom communities around the network environment^65^.

The contribution of this study is that it is the first network analysis study of anxiety and depression among Chinese college students majoring in computer science and related fields, and with a large sample size. The visualization and stability analysis of the network perspective helped us to obtain a clear and accurate symptom association structure that is easy to identify. This provides a new research paradigm for exploring anxiety and depression networks in diverse student populations, with a focus on emphasizing the relationship between core symptoms, symptom clusters, and potential comorbidities. It has been shown to help in intervening with symptoms of mental disorders in clinical practice through interconnections between a limited number of symptom modules^33,65^. Furthermore, the combination of network structure and community detection provided a more comprehensive understanding of symptom clustering and interactions within an anxiety-depression framework. In summary, the results of this study provide a feasible research paradigm for exploring potential mental health problems in student populations from diverse educational backgrounds.

There are still some limitations of this study that need to be noted. First, although the sample size was large, participant recruitment was limited to Henan Province due to funding, time, and resource constraints. While Henan Province is considered a representative educational region in terms of student population, future studies should expand the range of participants to the whole country. Second, the anxiety and depression scales of the GAD7 and PHQ9 were used only to obtain a means of individual self-assessment. Despite such scales are widely used, biases about individual differences in interpretation may still exist^30^. Third, cross-sectional studies limit causality in anxiety-depression networks. We have realized this limitation and have initiated follow-up studies incorporating additional dimensions and time sequences. Future studies should retain the directional structure advantage of network analysis, while also need more clinical and experimental data. Longitudinal study design and clinical trial-based symptom assessment will help enhance its applicability in clinical decision-making^65^. This integration may allow for a more robust evaluation of symptom dynamics, causality, and treatment feedback within the framework of symptom network analysis.

## Conclusion

In summary, through network analysis of anxiety and depression among Chinese college students majoring in computer science, we identified the connection between Nervousness and Uncontrollable worry is the strongest edge in the network. Three core symptoms with the highest node strength were concentration, fatigue and psychomotor problems. Three bridge symptoms with the highest bridge strength were irritability, feeling afraid and psychomotor problems. Four well-characterized symptom communities were identified under the detection of the network community algorithm, covering the core symptoms of anxiety, somatization and emotional symptoms of anxiety, the core symptoms of depression, and symptoms related to somatization manifestations in depressive symptoms. These findings are important for future interventions and amelioration of mental health issues for students with different majors and stressors. Moreover, this network analysis framework can be further extended to other high-stress groups to help clinicians and educational departments develop more comprehensive support policies and interventions.

## Supplementary Information

Below is the link to the electronic supplementary material.

Supplementary Material 1

## Data Availability

Data and core code can be obtained from the corresponding author upon reasonable request.
