# Supplementary material for: Identifying symptom communities and core symptoms in the anxiety-depression network among computer science students
Source: Sci Rep. 2026 Apr 8;16:11649. doi: 10.1038/s41598-026-39553-w (PMC13061885; doi:10.1038/s41598-026-39553-w)
Supplement: Supplementary file 1 — Supplementary Material 1 [file 41598_2026_39553_MOESM1_ESM.docx]

# Identifying Symptom Communities and Core Symptoms in the Anxiety-Depression Network among Computer Science Students: A Network Approach

Wei Yi^1^, Kun Yang^1^, Zhengfan Wei^1^, Mohd Mahzan Awang^2^, Wan Ahmad Munsif Wan Pa^2^, Yonglin Chen^2^, Meiyang Wang^3^, Shuoyu Jing^2*^

^*Corresponding author's e-mail: p119216@siswa.ukm.edu.my^

^1^College of Information Engineering, Zhengzhou University of Science and Technology, Zhengzhou, China.

^2^Faculty of Education, National University of Malaysia, Bangi, Malaysia.

^3^College of Clinic, Sanquan College of Xinxiang Medical University, Xinxiang, China.

**Author contributions**

W.Y and SY.J led the research, K.Y, ZF.W and YL.C were responsible for data collection, organization and cleaning. M.M.A and W.A.M.W.P supervised the research design and methodology of the article. All authors (W.Y, SY.J, K.Y, ZF.W, YL.C, M.M.A, W.A.M.W.P, MY.W) participated in the writing of the article's results, discussion, and first draft.

**Supplement Table S1**

**Table S1.** Partial correlation matrix

| **GAD1** | **GAD2** | **GAD3** | **GAD4** | **GAD5** | **GAD6** | **GAD7** | **PHQ1** | **PHQ2** | **PHQ3** | **PHQ4** | **PHQ5** | **PHQ6** | **PHQ7** | **PHQ8** | **PHQ9** |
| --- | --- | --- | --- | --- | --- | --- | --- | --- | --- | --- | --- | --- | --- | --- | --- |
| 0 | 0.20710102 | 0.180209541 | 0.006018438 | 0 | 0.013371811 | 0 | 0.0025112 | 0 | 0 | 0 | 0 | 0.004570903 | 0 | 0 | -0.012311979 |
| 0.20710102 | 0 | 0.046280331 | 0.001593046 | 0.096163953 | 0 | 0.021103581 | 0 | 0.023590654 | 0 | 0 | 0 | 0 | 0 | 0 | 0 |
| 0.180209541 | 0.046280331 | 0 | 0.107230925 | 0 | 0.028863837 | 0.049234474 | 0 | -0.003090104 | 0 | 0.019102452 | 0 | 0 | 0 | 0 | -0.017486208 |
| 0.006018438 | 0.001593046 | 0.107230925 | 0 | 0.197710638 | 0 | 0.025304883 | 0 | 0 | 0 | 0 | 0 | 0 | 0 | 0 | 0 |
| 0 | 0.096163953 | 0 | 0.197710638 | 0 | 0.131201744 | 0.02404241 | 0.028908314 | 0.014482955 | 0 | 0 | 0 | 0.00505359 | 0 | 0 | 0 |
| 0.013371811 | 0 | 0.028863837 | 0 | 0.131201744 | 0 | 0.126046846 | 0 | 0 | 0 | 0.026473686 | 0 | -0.029857872 | 0 | 0.127306003 | 0 |
| 0 | 0.021103581 | 0.049234474 | 0.025304883 | 0.02404241 | 0.126046846 | 0 | 0.033876324 | 0.076812821 | 0 | 0.006547182 | 0.006391821 | 0.057114049 | 0 | 0 | 0 |
| 0.0025112 | 0 | 0 | 0 | 0.028908314 | 0 | 0.033876324 | 0 | 0.042740161 | 0 | 0.050671497 | 0 | 0 | 0.185333852 | -0.014100314 | -0.02183619 |
| 0 | 0.023590654 | -0.003090104 | 0 | 0.014482955 | 0 | 0.076812821 | 0.042740161 | 0 | 0.029628623 | 0 | 0.024593758 | 0.047358195 | 0 | 0.084578055 | 0.114876862 |
| 0 | 0 | 0 | 0 | 0 | 0 | 0 | 0 | 0.029628623 | 0 | 0.20002854 | 0.058938326 | 0 | 0 | 0.005474891 | 0.012867464 |
| 0 | 0 | 0.019102452 | 0 | 0 | 0.026473686 | 0.006547182 | 0.050671497 | 0 | 0.20002854 | 0 | 0.102037905 | 0 | 0.092120364 | 0 | -0.024099751 |
| 0 | 0 | 0 | 0 | 0 | 0 | 0.006391821 | 0 | 0.024593758 | 0.058938326 | 0.102037905 | 0 | 0.046645704 | 0 | 0.007381232 | 0.093313995 |
| 0.004570903 | 0 | 0 | 0 | 0.00505359 | -0.029857872 | 0.057114049 | 0 | 0.047358195 | 0 | 0 | 0.046645704 | 0 | 0.125543411 | 0.066624466 | 0.096960545 |
| 0 | 0 | 0 | 0 | 0 | 0 | 0 | 0.185333852 | 0 | 0 | 0.092120364 | 0 | 0.125543411 | 0 | 0.129902419 | 0 |
| 0 | 0 | 0 | 0 | 0 | 0.127306003 | 0 | -0.014100314 | 0.084578055 | 0.005474891 | 0 | 0.007381232 | 0.066624466 | 0.129902419 | 0 | 0.076820674 |
| -0.012311979 | 0 | -0.017486208 | 0 | 0 | 0 | 0 | -0.02183619 | 0.114876862 | 0.012867464 | -0.024099751 | 0.093313995 | 0.096960545 | 0 | 0.076820674 | 0 |

**A Pseudo-Code Guide to Network Analysis**

1. Load symptom data matrix from Excel or CSV

→ Use read_excel() or read.csv()

data_matrix ← read_excel("your_file.xlsx")

2. Check and convert all variables to numeric (if needed)

→ Apply lapply() and as.numeric() across all columns

data_matrix[] ← lapply(data_matrix, as.numeric)

3. Compute Pearson correlation matrix

→ cor() function with method = "pearson"

cor_matrix ← cor(data_matrix, method = "pearson")

4. Convert to partial correlation matrix

→ Use cor2pcor() function (from psych or corpcor package)

partial_cor_matrix ← cor2pcor(cor_matrix)

5. Prepare node labels and color schemes

node_labels ← colnames(partial_cor_matrix)

node_colors ← Set colors by symptom group (e.g., anxiety vs depression)

6. Visualize network with qgraph (spring layout)

→ qgraph() with custom layout, labels, and visual parameters

qgraph_network ← qgraph(

partial_cor_matrix,

layout = "spring",

labels = node_labels,

color = node_colors,

vsize = 6,

edge.width = 1.5

)

7. Compute centrality metrics (strength, bridge strength)

→ Use centrality_auto() from qgraph

centrality_results ← centrality_auto(qgraph_network)

8. Compute expected influence (EI)

→ 1-step EI: sum of partial correlations

→ 2-step EI: matrix multiplication

expected_influence_1step ← colSums(partial_cor_matrix)

expected_influence_2step ← colSums(partial_cor_matrix %*% partial_cor_matrix)

9. Fit Mixed Graphical Model (MGM)

→ Use mgm() from mgm package

mgm_model ← mgm(data = data_matrix, type = rep("g", n), level = rep(1, n), k = 2)

10. Calculate node predictability

→ Use predict() with the MGM model

predictability_results ← predict(object = mgm_model, data = data_matrix)

11. Detect symptom communities (Spin Glass algorithm)

→ Convert partial matrix to igraph object using graph_from_adjacency_matrix()

→ Use cluster_spinglass() with adjusted gamma

g ← graph_from_adjacency_matrix(partial_cor_matrix, weighted = TRUE, mode = "undirected")

set.seed(1234)

spinglass_communities ← cluster_spinglass(g, gamma = 0.6)
